# Sex Differences in Music: A Female Advantage at Recognizing Familiar Melodies

**DOI:** 10.3389/fpsyg.2016.00278

**Published:** 2016-03-01

**Authors:** Scott A. Miles, Robbin A. Miranda, Michael T. Ullman

**Affiliations:** ^1^Brain and Language Laboratory, Department of Neuroscience, Georgetown University, WashingtonDC, USA; ^2^Interdisciplinary Program in Neuroscience, Georgetown University, WashingtonDC, USA; ^3^Infinimetrics Corporation, ViennaVA, USA

**Keywords:** music, music cognition, melody, declarative memory, recognition, sex differences, musical training, language

## Abstract

Although sex differences have been observed in various cognitive domains, there has been little work examining sex differences in the cognition of music. We tested the prediction that women would be better than men at recognizing familiar melodies, since memories of specific melodies are likely to be learned (at least in part) by declarative memory, which shows female advantages. Participants were 24 men and 24 women, with half musicians and half non-musicians in each group. The two groups were matched on age, education, and various measures of musical training. Participants were presented with well-known and novel melodies, and were asked to indicate their recognition of familiar melodies as rapidly as possible. The women were significantly faster than the men in responding, with a large effect size. The female advantage held across musicians and non-musicians, and across melodies with and without commonly associated lyrics, as evidenced by an absence of interactions between sex and these factors. Additionally, the results did not seem to be explained by sex differences in response biases, or in basic motor processes as tested in a control task. Though caution is warranted given that this is the first study to examine sex differences in familiar melody recognition, the results are consistent with the hypothesis motivating our prediction, namely that declarative memory underlies knowledge about music (particularly about familiar melodies), and that the female advantage at declarative memory may thus lead to female advantages in music cognition (particularly at familiar melody recognition). Additionally, the findings argue against the view that female advantages at tasks involving verbal (or verbalizable) material are due solely to a sex difference specific to the verbal domain. Further, the results may help explain previously reported cognitive commonalities between music and language: since declarative memory also underlies language, such commonalities may be partly due to a common dependence on this memory system. More generally, because declarative memory is well studied at many levels, evidence that music cognition depends on this system may lead to a powerful research program generating a wide range of novel predictions for the neurocognition of music, potentially advancing the field.

## Introduction

Sex differences have been observed in various cognitive domains. For example, it has been suggested that boys and men have advantages at aspects of visuospatial cognition, while girls and women are better at aspects of verbal cognition (Kimura, 1999; Halpern, 2013). Sex differences in a variety of other domains have also been examined, though inconsistent findings and variability in the magnitude of the effects have led to questions about the existence of sex differences in cognition (Hyde, 2005).

There has been little examination, however, of sex differences in the cognition of music. This seems somewhat surprising, given the surge of research on music cognition in recent decades (Levitin and Tirovolas, 2009; Tirovolas and Levitin, 2011), as well as the apparent sex differences found in verbal cognition. Recent evidence suggests that the processing of language and music may be subserved by at least partially overlapping neural substrates (Patel, 2003; Brown et al., 2006). It is possible that some of the sex differences observed in language are driven by sex differences in these common substrates, suggesting they may extend to music cognition as well.

A relatively small number of neurocognitive studies have examined behavioral sex differences in aspects of music cognition. These studies have focused mainly on the low-level perception of single auditory events, such as those involved in spontaneous and click-evoked otoacoustic emissions (Snihur and Hampson, 2011), transient evoked otoacoustic emissions (Cassidy and Ditty, 2001), and pitch memory (Gaab et al., 2003). Music is, however, a complex phenomenon, consisting of several such events unfolding and interacting in time. It is possible that this focus on the low-level perception of single auditory events has left undetected behavioral sex differences in higher-level aspects of music cognition.

A useful distinction can be made between two higher level aspects of music cognition: knowledge of the general patterns of a musical system, often referred to as knowledge of musical syntax (Koelsch and Friederici, 2003; Koelsch et al., 2013; Sammler et al., 2013; Matsunaga et al., 2014) or *schematic* knowledge (Bharucha, 1994; Tillmann and Bigand, 2001; Huron, 2006); and knowledge of the idiosyncratic representations in music, such as of specific melodies, sometimes referred to as *veridical* knowledge (Bharucha, 1994; Huron, 2006). It has been proposed that much of the aesthetic value of music comes from the adherence to and violation of expectations generated by each of these two types of knowledge (Bharucha, 1994). It has also been proposed that the two types of knowledge can be dissociated, and may depend on different memory systems in the brain (Huron, 2006; Miranda and Ullman, 2007). This proposal is supported by an event-related potential (ERP) study demonstrating a double dissociation between the processing of violations of musical syntax and violations of familiar melodies, which involve idiosyncratic representations (Miranda and Ullman, 2007). Given these dissociations, it is possible that sex differences may be found in either syntactic (schematic) or idiosyncratic (veridical) aspects of music cognition, but not in both.

We are aware of two studies that have examined behavioral sex differences in higher-level aspects of music cognition (Koelsch et al., 2003a,b). Both of these focused on musical syntax, probing responses to violations of syntactic expectations. Though sex differences in electrophysiological brain responses (as measured by ERPs) were observed in both studies, neither found sex differences in performance. Of course, such null effects could be due to many factors. The possibility remains, however, that there are indeed performance advantages for one sex over the other in tasks of higher-level music cognition, but that these involve knowledge of idiosyncratic aspects of music rather than knowledge of musical syntax.

Indeed, as we shall see, some previous evidence suggests that knowledge regarding specific aspects of melodies is stored, at least in part, in declarative memory, a general-purpose memory system that is critical for learning idiosyncratic information in general, including in language. Crucially, declarative memory also shows sex differences, in particular a female advantage, including in the recognition of previously learned idiosyncratic verbal material such as vocabulary items. Thus it is possible that this female advantage might extend to aspects of music cognition that depend on this memory system. Specifically, a female advantage may be expected in the recognition of familiar melodies, which involve idiosyncratic representations. We tested this prediction in the present study by examining the performance of men and women in a familiar melody recognition task.

In the remainder of the Introduction, we first briefly summarize the nature of declarative memory and evidence suggesting sex differences in this system. We then lay out the evidence suggesting that in music cognition, the storage and retrieval of knowledge about specific melodies depends, at least in part, on declarative memory. Finally, we summarize the present study.

### Declarative Memory: Overview and Sex Differences

Declarative memory is quite well understood (for reviews, see Ullman, 2004, 2016; Henke, 2010; Squire and Wixted, 2011; Eichenbaum, 2012; Cabeza and Moscovitch, 2013). As its name suggests, this memory system underlies the learning, storage, and retrieval of explicit knowledge, which is available to conscious awareness – although increasing evidence indicates that it also subserves implicit knowledge (Henke, 2010; Ullman, 2016). The system is rooted in the hippocampus and other medial temporal lobe structures. These structures are critical for the learning and consolidation of new knowledge. The subsequent storage of much of this knowledge, however, eventually relies largely on neocortical regions, especially in the temporal lobes. Declarative memory may be specialized for learning arbitrary bits of information and binding them together (Henke, 2010; Squire and Wixted, 2011). Indeed, the system may be necessary for learning such idiosyncratic information. This may help explain evidence that damage to the declarative memory system can severely impair or even prevent the learning of knowledge about words and other idiosyncratic information (Squire and Wixted, 2011; Ullman, 2016).

Increasing evidence suggests a female advantage at declarative memory, including in idiosyncratic aspects of language (for a discussion and review of the literature, see Ullman et al., 2008). Studies have shown female advantages for a wide variety of episodic memory tasks (which crucially depend on declarative memory), including those testing verbal material, landmarks, objects, object locations, novel faces, and complex abstract patterns (Ullman et al., 2008). A female advantage has also been reported for word learning (Kaushanskaya et al., 2011) and for the retrieval of well-established (previously learned) knowledge, including in tests of vocabulary, lexical retrieval, and verbal fluency (Ullman et al., 2008). These behavioral female advantages are consistent with anatomical sex differences (Ullman et al., 2008). For example, the hippocampus seems to develop at a faster rate, with respect to the rest of the brain, in girls than in boys between the ages of one and sixteen (Pfluger et al., 1999). The behavioral and anatomical sex differences may be at least partly mediated by estrogen, which is found in higher levels in girls and (pre-menopausal) women than in boys and men (Wilson et al., 1998), and affects declarative memory and hippocampal structure and function, through both organizational effects *in utero* and activation effects later on (Phillips and Sherwin, 1992).

Given the dependence of idiosyncratic (and other) aspects of language on declarative memory (Ullman, 2004, 2016), many if not most of the previously reported sex differences in language may in fact be explained by broader, domain-independent sex differences in the declarative memory system (Ullman, 2004, 2016; Ullman et al., 2008). Accordingly, the female advantage at the storage and retrieval of idiosyncratic representations may extend beyond previously studied verbal and non-verbal domains and functions to music cognition – in particular to the storage and retrieval of knowledge about specific melodies.

### Melodies, Declarative Memory, and Expected Sex Differences

As we have seen, the cognition of music, like that of language, requires the memorization of specific, idiosyncratic representations, including of familiar melodies. Melodies contain specific sequences of notes that must be veridically learned, even though the sequences are also schematically constrained by the syntax of a musical system – much like words involve particular sequences of phonemes that are also constrained by the rules of phonotactics. Given that declarative memory seems to underlie the learning and storage of knowledge about words, and more generally may be necessary for learning arbitrary bits of information and binding them together, it may be expected that this system is also critical for learning idiosyncratic representations in music, including knowledge about specific melodies.

Some evidence already suggests that this may be the case. In an electrophysiological study, an ERP component characterized as an N400 was observed in response to expectation violations resulting from altered notes within melodies that were well known (and thus likely to be familiar to participants), but not to violations of notes within novel melodies (Miranda and Ullman, 2007). N400s, which originate in part in the medial temporal lobe (McCarthy et al., 1995; Meyer et al., 2005), and are found in response to a variety of lexical stimuli, as well as to idiosyncratic non-verbal stimuli such as objects and faces (Kutas and Federmeier, 2011), have been linked to declarative memory (Ullman, 2001, 2016). The findings of the music ERP study (Miranda and Ullman, 2007) thus suggest that, like knowledge of these various types of non-musical idiosyncratic information, knowledge about familiar melodies may also be stored in and retrieved from declarative memory.

Given the female advantages observed in other tasks involving declarative memory, including in both the learning of new knowledge and the retrieval of previously learned information, such advantages might also extend to knowledge of idiosyncratic representations in music, including of familiar melodies. We thus predicted a female advantage at recognizing familiar melodies.

### The Present Study

To test this prediction we examined the recognition of well-known melodies in adults. We focused on the recognition of already-known melodies, rather than the learning of new melodies, because previous evidence suggests that consolidation – even over the course of months or longer – can significantly affect outcomes (Marshall and Born, 2007; Morgan-Short et al., 2012).

Healthy men and women were presented with both well-known and novel melodies. Participants were asked to indicate as quickly and accurately as possible during the presentation of each melody whether they were familiar with it. Response time (RT) as well as accuracy measures were obtained. RTs typically provide greater variability than accuracy, and minimize the likelihood of ceiling effects. In addition, some previous evidence suggests that the time element may be important in revealing the hypothesized female advantages (Walenski et al., 2008).

We examined both musicians and non-musicians. This allowed us to test how broadly the findings may hold across musical training. Testing across musicians and non-musicians is also important because previous studies examining neural sex differences have found interactions between sex and musical training (Evers et al., 1999; Hutchinson et al., 2003). Musicians might be expected to show stronger representations of familiar melodies simply due to greater exposure (Besson and Faïta, 1995). It is also plausible that members of either sex might have had greater previous exposure to the well-known melodies than members of the other sex. To attempt to address these issues, after each of their timed recognition responses, participants were asked to report a familiarity rating for the melody. By covarying out these ratings in our analyses, we were able to test whether any group differences in performance held even when familiarity was held constant.

All of the stimuli were presented instrumentally. However, since many of the melodies in the study are commonly associated with lyrics, any observed female advantages could in principle be due to advantages in the verbal domain, rather than in familiar melody recognition itself. We therefore separated the melodies into those that are or are not associated with lyrics, to be able to test whether any sex differences might hold across both.

Finally, it is possible that any observed sex differences in the recognition of melodies might be due to sex differences in basic motor processes, rather than differences in music cognition. To help rule out this possibility, we also gave participants a control task, in which they were asked to respond to single tones as quickly as possible. If the sex differences were due to lower-level motor processes, any differences in the experimental task might also be reflected in the results of the control task.

Overall, given the hypothesis that the female advantage in declarative memory should extend to knowledge about familiar melodies, we predicted that women would show faster and perhaps more accurate recognition of well-known melodies than men. Moreover, we expected this advantage to hold broadly, over both musicians and non-musicians, and across melodies with and without lyrics, and that the advantage would not be fully explained by sex differences in familiarity or in basic motor processes.

## Materials and Methods

### Participants

Participants were right-handed native speakers of American English. They had no known developmental, neurological, or psychiatric disorders. Since familiarity with the well-known melodies used in this study is largely culture-dependent, we selected only participants who had not lived outside of the United States for more than 6 months before the age of 18. Research methods were approved by the Institutional Review Board at Georgetown University. All participants gave written informed consent and received monetary compensation for their participation.

Two groups of participants were tested: 24 men and 24 women. Half of the participants within each group were musicians and half were non-musicians. The musicians had at least 4 years of formal musical training, which was defined as private instrument or voice lessons, or participation in a musical ensemble. The non-musicians had 1 year or less of formal musical training. In our initial analysis of RTs to well-known melodies (described below), we found that two of the participants were outliers (one female musician and one female non-musician), each having a mean RT greater than two standard deviations from the mean RT for their respective participant subgroup. The data from these two participants were excluded and replaced with data from two newly tested participants: one female musician and one female non-musician.

The final two groups of participants therefore also consisted of 24 participants each. **Table 1** shows information for each of the four 12-member subgroups regarding age, years of education, handedness (Oldfield, 1971), years of formal musical training, and (for the musicians only) age when formal musical training began, number of years since last formal musical training, number of instruments played (including voice), and number of participants who still regularly played an instrument or sang at the time of testing. Results from 2 × 2 analyses of variance (ANOVAs), with the factors Sex (male/female) and Musical Training (musician/non-musician), confirmed that the four subgroups did not differ significantly in age [Sex: *F*(1,44) = 0.20, *p* = 0.656, Musical Training: *F*(1,44) = 0.20, *p* = 0.656, Sex by Musical Training: *F*(1,44) = 0.04, *p* = 0.848], years of education [Sex: *F*(1,44) = 0.34, *p* = 0.561, Musical Training: *F*(1,44) = 0.18, *p* = 0.677, Sex by Musical Training: *F*(1,44) = 0.03, *p* = 0.868)], or handedness [Sex: *F*(1,41) = 0.03, *p* = 0.870, Musical Training: *F*(1,41) = 0.46, *p* = 0.500, Sex by Musical Training: *F*(1,41) = 3.27, *p* = 0.078; note that values were missing from three participants; see **Table 1**]. Importantly, the male and female musicians did not differ significantly in the number of years of formal musical training [*t*(22) = 0.46, *p* = 0.653]; the same was true for male and female non-musicians [*t*(22) = 0.67, *p* = 0.511]. Furthermore, there were no significant differences between male and female musicians regarding the age when musical training began [*t*(22) = 1.47, *p* = 0.156], the number of years since last formal musical training [*t*(22) = 1.11, *p* = 0.278], the number of instruments (including voice) played by each participant [*t*(22) = 0.99, *p* = 0.335], or the number of participants who regularly played a musical instrument or sang at the time of the experiment [*t*(22) = 0.80, *p* = 0.430].

**Table 1:** Participant information on age, education, and musical training.

	Age in years	Years of education	Handedness	Years of formal musical training	Age when musical training began	Years since last musical training	Number of instruments played (including voice)	Number of participants currently engaged in instrument or vocal activities
Male musicians	20.2 (2.0)	14.4 (1.5)	93.6 (8.1)	7.8 (2.4)	8.8 (2.7)	3.2 (3.2)	2.3 (0.8)	6
Female musicians	20.3 (1.8)	14.7 (1.8)	88.3 (12.7)	8.3 (2.6)	7.3 (2.0)	4.6 (3.1)	1.9 (1.2)	4
Male non-musicians	20.3 (3.0)	14.1 (2.1)	90.0 (12.6)	0.3 (0.4)	NA	NA	NA	NA
Female non-musicians	20.8 (2.0)	14.5 (1.5)	96.4 (9.2)	0.2 (0.4)	NA	NA	NA	NA


*All values are presented as means (with standard deviations), except for the number of participants currently engaged in instrument or vocal activities. Handedness values were computed from the Edinburgh Handedness Inventory (Oldfield, 1971), with a value of 100 indicating maximally right handed; the means and standard deviations for male musicians, male non-musicians, and female non-musicians are based on data from 11 rather than 12 participants, since Edinburgh Handedness Inventory data were missing from one participant in each of these subgroups. NA, not applicable. See main text for details.*

### Stimuli

The musical stimuli consisted of 260 melodies ranging from 4.1 to 15.8 s in length (mean = 8.2 s, *SE* = 0.17). The stimuli were created in MIDI format using Finale Version 3.5.1 (Coda Music) and then converted to WAV files with a “grand piano” sound font using MidiSyn Version 1.9 (Future Algorithms). All melodies were in the key of C-major or C-minor. Half of the melodies (130) were segments from well-known tunes (see Appendix, in Supplementary Material), including traditional, folk, children’s, patriotic, holiday, classical, and pop music, as well as themes from movies, television, and Broadway musicals. The other half (130) were novel melodies composed by one of the authors (RM). The novel melodies served only as foils for the familiar melody recognition task, and are not reported or analyzed here. Each novel melody was composed to correspond to one of the well-known melodies. More specifically, the tempo and implied harmony (possible accompanying chords that are not present, but strongly suggested by the sequence of notes in the melody) of each novel melody were identical to those of its corresponding well-known melody; moreover, pitch range was closely matched. Distinctive rhythms were slightly altered in some of the novel melodies in order to minimize false recognition of these melodies based on rhythm. False recognition of novel melodies based on rhythm was not of great concern, in any case, since pitch structure has been found to be a better cue for the recognition of melodies than rhythmic structure (Hébert and Peretz, 1997).

### Experimental Task

For the purpose of counterbalancing, the 260 melodies were presented over the course of three runs, with each run containing a similar number (43 or 44) of well-known and novel melodies. Any given well-known melody and its matched novel melody were always presented in separate runs. The order of the three runs was counterbalanced across participants, such that for every six participants in each of the subgroups, the runs were presented in all possible orders. The presentation order of well-known and novel melodies was randomized within each run for each participant. Completion time for each run was approximately 15 min.

Melodies were presented on a laptop computer running Microsoft Windows, using Meds 2002 Revision B-1 (UCLA, Los Angeles). Participants were instructed to listen to each melody and to press the space bar as soon as the melody sounded familiar. If the melody was not recognized as familiar, the participant was instructed to wait until the end of the melody and then press the space bar to advance (only the keystrokes that occurred prior to the end of the melody were analyzed as responses). The full melody was presented regardless of when the space bar was pressed.

Immediately after the melody was completed and the space bar was pressed (whichever came last), the participant was prompted to rate the familiarity of the melody from 0 to 100, with 0 being most familiar (we selected this rating scale due to software constraints). Prior to testing, each participant received written instructions specifying that a rating of “0” should indicate “very familiar” melodies that the participant would be able to hum along with, whereas a rating of “100” should indicate melodies that were not familiar at all to the participant. The rating scale was shown on the screen as a horizontal scroll bar with “0” on the left and “100” on the right, with the words “Familiar” and “Unfamiliar” positioned under the left and right sides of the bar, respectively. The participant used a mouse to move a marker on the scroll bar to select the rating of his or her choice. As expected, the participants were indeed broadly familiar with the well-known melodies (mean rating of 17.9, *SD* = 9.0).

All participants were instructed to press the space bar with the left hand and to operate the mouse with the right hand, keeping the left hand just over the space bar at all times in order to minimize RTs. Before starting the experiment, each participant was given a practice run that included eight melodies, four of which were well known and four of which were novel.

### Control Task

After five participants had been tested on the experimental task, a control task was added to determine whether possible RT differences between participant groups could be attributed to group-wide differences in basic motor functions. The remaining participants (9 male musicians, 11 male non-musicians, 11 female musicians, and 12 female non-musicians) were given this task after completing all three runs of the experimental task. The control task included 20 tones of different pitches, each 500 ms long, presented at staggered intervals (between 0.3 and 2.1 s) after the participant’s previous response. Each participant was instructed to press the space bar with the left hand as soon as s/he heard a tone. Analysis of these RTs for each participant group revealed that three participants (one male musician, one female musician, and one female non-musician) were outliers, each having a mean RT greater than two standard deviations from the mean RT of their corresponding participant subgroup. Data from these participants were excluded from analyses of this task, and the data from the remaining eight male musicians, 11 male non-musicians, 10 female musicians, and 11 female non-musicians were subjected to full analysis.

## Results

### Response Times to Well-Known Melodies

Means for the recognition RTs to well-known melodies – that is, the latencies of responses registered during the presentation of these melodies – are shown for each of the four subgroups in the first column of **Table 2.** Prior to analysis, these were natural log transformed. Next, we eliminated very slow trials, which might result from diminished attention to the task. Specifically, for each participant, we eliminated trials with RTs that were greater than two standard deviations (SDs) above that participant’s mean. This resulted in the exclusion of a total of 2.69% of responses as outliers (135 out 5,012 correct responses to well-known melodies). To maintain an overall Type I Error probability of 0.05, we applied the Bonferroni correction: since six AN(C)OVAs were performed on the data from the experimental task, the significance level was set at 0.05/6 = 0.0083.

**Table 2:** Performance at the melody recognition and control tasks for each subgroup of participants.

	Recognition RTs to well-known melodies (ms)	RTs to tones in the control task (ms)	Recognition accuracy for well-known melodies
Male musicians	3607 (430)	404 (35)	81.2% (9.9%)
Male non-musicians	4037 (729)	352 (13)	70.5% (20.0%)
Female musicians	3169 (451)	390 (24)	88.7% (8.5%)
Female non-musicians	3483 (305)	386 (35)	81.5% (9.8%)


*Means (and standard deviations), computed over participants’ untransformed data (i.e., without natural log or arcsine transformations). ms, milliseconds.*

**FIGURE 1:**
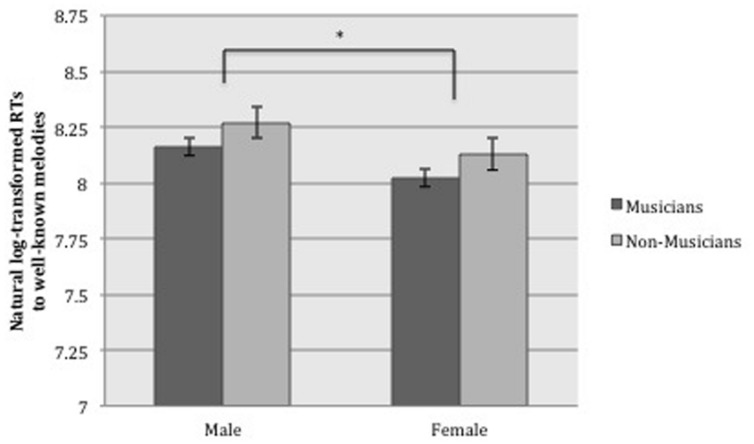
**Mean recognition response times (log-transformed) and standard errors for well-known melodies, showing the main effect of sex (^∗^*p* < 0.0083, based on Bonferroni correction for multiple comparisons)**.

These transformed and filtered RTs were then entered into a 2 × 2 ANOVA, with Sex (male/female) and Musical Training (musician/non-musician) as between-group factors. The ANOVA yielded a significant (i.e., following Bonferroni correction) main effect of Sex [*F*(1, 44) = 11.09, *p* = 0.002, $\eta_{p}^{2}$ = 0.201], with a large effect size (Cohen, 1988), indicating that women were significantly faster than men at responding to well-known melodies; see **Figure 1.** There was no significant main effect of Musical Training [*F*(1,44) = 6.27, *p* = 0.016, $\eta_{p}^{2}$ = 0.125] (though there was a tendency for musicians to respond faster than non-musicians), nor was there any interaction between Sex and Musical Training [*F*(1,44) = 0.001, *p* = 0.981, $\eta_{p}^{2}$ < 0.001], suggesting that the female advantage held similarly for musicians and non-musicians.

#### Familiarity as a Possible Confound

There was a significant correlation between participants’ mean recognition RTs and their mean familiarity ratings for well-known melodies [*r*(46) = 0.62, *p* < 0.001]. Accordingly, it is possible that women were faster at responding to well-known melodies simply because they were more familiar with the melodies, as compared to men. If this were the case, then including familiarity ratings as a covariate in the analysis would be expected to eliminate the finding of sex differences in RTs.

To examine this issue, a 2 (Sex) × 2 (Musical Training) analysis of covariance (ANCOVA) was performed on recognition RTs, with the covariate constituting each participant’s mean familiarity rating over all of the well-known melodies. The pattern of significance was identical to that described above. The analysis yielded a main effect of Sex [*F*(1,44) = 9.79, *p* = 0.003, $\eta_{p}^{2}$ = 0.185], with a large effect size, but there was no significant effect of Musical Training [*F*(1,44) = 4.04, *p* = 0.051, $\eta_{p}^{2}$ = 0.086], nor an interaction between Sex and Musical Training [*F*(1,44) = 1.22, *p* = 0.276, $\eta_{p}^{2}$ = 0.028]. These results suggest that the effects of Sex on recognition RTs could not be explained by group differences in familiarity. [Note that an ANOVA on mean familiarity ratings over all well-known melodies revealed no effects of Sex (*F*(1,44) = 1.57, *p* = 0.217, $\eta_{p}^{2}$ = 0.034) or Musical Training (*F*(1,44) = 1.96, *p* = 0.168, $\eta_{p}^{2}$ = 0.043), nor an interaction between them (*F*(1,44) = 2.23, *p* = 0.142, $\eta_{p}^{2}$ = 0.048)].

#### Response Bias as a Possible Confound

It is possible that the observed sex difference in RTs could be explained by differential response biases between the men and women. In particular, if the women had a greater tendency to respond with a recognition key press to all stimuli (novel as well as well-known melodies), this might account for their RT advantage in recognizing familiar melodies.

To address this concern, we performed a 2 (Sex) × 2 (Musical Training) ANOVA on bias scores [*c* = -0.5^∗^z(Hit rate) + z(False Alarm rate)]. This analysis revealed no main effects and no interaction [Sex: *F*(1,44) = 2.19, *p* = 0.146, $\eta_{p}^{2}$ = 0.048; Musical Training: *F*(1,47) = 2.95, *p* = 0.093, $\eta_{p}^{2}$ = 0.063; Sex by Musical Training: *F*(1,44) = 0.12, *p* = 0.726, $\eta_{p}^{2}$ = 0.003], suggesting that there were no differences between the groups in their response biases. This in turn suggests that the advantage for women over men at RTs in recognizing familiar melodies could not be explained by group differences in response biases.

#### Verbal Ability as a Possible Confound

As mentioned above, although the musical stimuli were presented without lyrics, many of the well-known melodies used in the study are often associated with lyrics. Thus, it might be argued that the female participants’ speed advantage at recognizing familiar melodies may have been specifically due to faster RTs for those melodies associated with lyrics, which women recognized more quickly because of their verbal associations. On this view, the sex differences observed here might be explained by a female advantage at processing verbal information, rather than an advantage at recognizing purely musical aspects of familiar melodies. If this were the case, we might expect to see an interaction between the factors of Sex and “Lyricness” (i.e., whether or not melodies are associated with lyrics). On the other hand, no such interaction would be expected if the sex difference held similarly across melodies that are associated with lyrics and those that are not.

To examine this issue, we first assessed each well-known melody’s association with lyrics by testing six native speakers of American English (four women, two men), ages 19–36 (mean = 23.8 years), with 1–14 years of musical training (mean = 9.2 years), none of whom had lived outside the United States for more than 6 months before age 18. None of these six participants were included in the larger experiment. The participants listened to all of the 130 well-known melodies. After each melody, they were presented with two questions, to which they responded “Yes” or “No.” The questions were presented, one after another, on a computer screen: “(1) Are you familiar with this melody?” and “(2) Do you associate this melody with any lyrics?” For the second question, participants were instructed to answer “Yes” to any melody for which they thought they knew either the actual lyrics or any other (informal) lyrics (e.g., any lyric that they had ever heard or sung with that particular melody). To determine the strength of the association between each melody and its possible lyrics, a “lyric familiarity” score was calculated as the percentage of participants who associated lyrics with the melody, only out of those participants who were familiar with the melody itself (since unfamiliarity with a melody inevitably resulted in unfamiliarity with that melody’s lyrics). Of the 130 melodies, 105 received a lyric familiarity score of 50% or higher (mean = 86.0%) and were considered “lyrics” melodies, while the remaining 25 melodies received a score below 50% (mean = 8.6%) and were considered “no-lyrics” melodies.

We then performed an ANOVA with the between-group factors Sex and Musical Training, and the within-group factor Lyricness (lyrics/no-lyrics melodies). This yielded a main effect of Sex [*F*(1,42) = 8.87, *p* = 0.005, $\eta_{p}^{2}$ = 0.168] as well as of Musical Training [*F*(1,42) = 7.78, *p* = 0.008, $\eta_{p}^{2}$ = 0.150], both with large effect sizes, but no interaction between Sex and Musical Training [*F*(1,42) = 0.05, *p* = 0.833, $\eta_{p}^{2}$ = 0.001]. Importantly, there was no significant main effect of Lyricness [*F*(1,42) = 5.450, *p* = 0.024, $\eta_{p}^{2}$ = 0.110], nor any significant interactions between Sex and Lyricness [*F*(1,42) = 0.930, *p* = 0.340, $\eta_{p}^{2}$ = 0.021], between Musical Training and Lyricness [*F*(1,42) = 0.390, *p* = 0.536, $\eta_{p}^{2}$ = 0.009], nor among Sex, Musical Training, and Lyricness [*F*(1,42) = 0.532, *p* = 0.470, $\eta_{p}^{2}$ = 0.012]. This analysis suggests that the RT advantage for women at the recognition of familiar melodies held similarly for melodies that were associated with lyrics and those that were not.

#### Basic Motor Processes as Possible Confounds

To test for the possibility that sex differences in basic motor processes could account for the women’s RT advantage over men, we administered a control task (see Materials and Methods for details, and **Table 2** for mean RTs by subgroup). Prior to analyses, the RTs were natural log transformed. Next, negative RTs (1.9% of all responses) resulting from premature responses were excluded from analysis. There were no very slow RTs (RTs greater than two SDs above each participant’s mean), so none were eliminated.

The 2 (Sex) × 2 (Musical Training) ANOVA on these RTs yielded no main effects of Sex [*F*(1,36) = 0.094, *p* = 0.760, $\eta_{p}^{2}$ = 0.003] or of Musical Training [*F*(1,36) = 0.778, *p* = 0.383, $\eta_{p}^{2}$ = 0.021], and no interaction between them [*F*(1,36) = 0.736, *p* = 0.396, $\eta_{p}^{2}$ = 0.020]. This suggests that the group differences in recognition RTs to well-known melodies are not likely to be explained by group differences in basic motor processes (at least those measured by this task).

### Accuracy

To examine whether the findings of a female advantage might extend beyond RTs, we also examined accuracy. Each participant’s percentage of correct recognition responses to all well-known melodies constituted the dependent variable in this analysis; see **Table 2.** These percentages were arcsine-transformed prior to analyses. A 2 (Sex) × 2 (Musical Training) ANOVA revealed no significant main effects, that is, neither of Sex [*F*(1,47) = 6.755, *p* = 0.013, $\eta_{p}^{2}$ = 0.132], nor of Musical Training [*F*(1,47) = 6.189, *p* = 0.017, $\eta_{p}^{2}$ = 0.123], nor an interaction between them [*F*(1,47) = 0.008, *p* = 0.928, $\eta_{p}^{2}$ < 0.001].

## Discussion

This study examined the prediction that women would have an advantage at recognizing familiar melodies, as compared to men. Indeed, women were significantly faster than men at recognizing familiar melodies, based on a Bonferroni corrected significance level. This sex difference yielded a large effect size (defined as $\eta_{p}^{2}$ ≥ 0.138; Cohen, 1988). The result held across musicians and non-musicians, as reflected by the absence of an interaction between sex and musicianship.

Unlike in the case of recognition RTs, we did not find a significant female advantage in our measure of accuracy, after correcting for multiple comparisons. However, as discussed above, accuracy is a less sensitive indicator of performance than RT. Perhaps for this reason, a female advantage was found for RTs but not accuracy in a recent study of lexical retrieval (Walenski et al., 2008). Indeed, it is possible that women are more accurate than men in their familiarity recognition responses, but our sample sizes (two groups of 24 participants each) were not large enough to demonstrate this effect. The finding of a significant female advantage in accuracy prior to correction for multiple comparisons is consistent with this view – especially since Bonferroni correction is quite conservative.

The female RT advantage was not explained by a number of potentially confounding factors. First, there were no significant group differences in various demographic variables that might have otherwise accounted for the observed advantages. The four subgroups (male musicians, male non-musicians, female musicians, and female non-musicians) did not differ in age, years of education, or handedness. Additionally, the male and female musicians did not differ in years of formal musical training, and likewise for the male and female non-musicians. The male and female musicians also did not differ regarding the age when their musical training began, the years since their last musical training, the number of instruments played (including voice), or the number of participants in each subgroup who were currently engaged in instrumental or vocal activities. Second, the advantages were not explained by group differences in familiarity with the well-known melodies. It might be suggested that the women were faster at recognizing well-known melodies because they were simply more familiar with the melodies than the men. However, the female advantage was observed even when familiarity ratings were covaried out. Third, since there were no group differences or interactions on bias scores, group differences in bias are also not likely to explain the observed female advantage. Fourth, the advantages could not be fully accounted for by associations between the melodies and lyrics. It might be argued that a female advantage in the verbal domain could explain the sex difference observed here, rather than an advantage in the recognition of familiar melodies *per se*. In particular, since quite a few of the melodies in the study are associated with lyrics, it might have been the case that the female advantage held only or mainly for these items. However, there were no significant interactions between lyricness and sex, suggesting the speed advantage for women held across melodies that are and are not commonly associated with lyrics. This in turn suggests that the findings cannot be explained by a female advantage purely in the verbal domain. Fifth, it is not likely that group differences in basic motor processes accounted for the female advantage in melody recognition, since there were no significant differences between the groups in performance during a simple tone detection control task. This suggests that at least the basic motor processes examined in this task did not differ between the groups, and thus were not likely to have explained the observed differences in melody recognition.

We suggest instead that the sex differences in recognition RTs are at least partly explained by the previously reported female advantage at declarative memory. As discussed in Section “Introduction,” this advantage has been found not only for learning new material, but also for the retrieval of previously learned material, as was tested in the present study. Together with independent electrophysiological evidence suggesting that the processing of familiar melodies depends at least in part on declarative memory (see Introduction, and Miranda and Ullman, 2007), the data from the present study suggest that the female advantage at declarative memory may indeed extend to music cognition, in particular to the retrieval of stored knowledge about melodies. However, given that this is the first study to examine sex differences in familiar melody recognition, some caution in interpreting the findings is warranted; see Section “Limitations and Future Studies” below for further discussion.

The claim that knowledge about familiar melodies depends on declarative memory does not presuppose that this is the only memory or other cognitive system involved in the learning, storage, or retrieval of such knowledge. For example, attention and working memory systems may be expected to play roles, at least in part because of their interactions with the declarative memory system for learning and retrieval (Ullman, 2004, 2016).

A role for declarative memory in stored knowledge of melodies also would not preclude additional roles for this system in music cognition. One interesting possibility is that declarative memory might, to some extent, play redundant roles with procedural memory in certain aspects of music cognition – for example, in learning and processing syntactic (schematic) knowledge, that is, knowledge about the regularities of musical systems. Increasing evidence suggests that such redundancy between declarative and procedural memory exists for language and other domains (Ullman, 2004, 2016). For example, individuals or groups with declarative memory advantages, or with deficits of procedural memory, appear to rely more on declarative memory, relative to procedural memory, for various grammatical functions (Ullman and Pullman, 2015; Ullman, 2016). Of particular interest here, girls and women seem to rely more on declarative memory than boys and men for aspects of grammar, likely due in part to the female advantages at declarative memory (Ullman et al., 2008; Ullman, 2016). It is plausible that such a sex difference might be found analogously for syntactic aspects of music cognition. Intriguingly, two studies have reported more bilateral negativities in girls and women than boys and men in response to syntactic anomalies within musical stimuli (Koelsch et al., 2003a,b). Although these negativities had primarily anterior distributions, their bilaterality suggests the possibility that they may be related to N400s, consistent with a greater dependence of musical syntactic processing on declarative memory in females than males. Indeed, such redundancy is consistent with the lack of sex differences in performance reported in these studies, since the errors may be processed equally well in the two systems (Ullman, 2004, 2016). However, this interpretation of these studies should be treated with caution, and future research is needed.

Although the goal of the present study was to test sex differences in melody recognition, and the observed female advantage was indeed the most robust effect, an advantage of musicians over non-musicians was also found. Musicians showed a significant (i.e., following Bonferroni correction) RT advantage in the analysis with lyricness as a factor, as well as RT and accuracy advantages that were significant or borderline significant prior to Bonferroni correction, in other analyses. The cause of this apparent effect is not entirely clear. One possibility is that musicians simply have greater familiarity with the melodies. Another possibility is that the training involved in learning to perform music results in improvements in declarative memory. Indeed, some evidence hints at declarative memory improvements from other types of training (Draganski et al., 2006; Woollett and Maguire, 2011). Alternatively (or in addition), perhaps individuals with better declarative memory (and maybe other advantages as well) are more likely to become musicians, or to stick with musical training. Finally, the fact that a significant musician advantage only emerged in the analysis with lyricness may be attributed to a reduction of the error term in this analysis due the inclusion of this factor. Future studies examining the apparent musician advantage at familiar melody recognition seem warranted.

### Implications

The present study has implications for various disciplines and endeavors. In the domain of music cognition, together with the ERP results of Miranda and Ullman (2007), it provides evidence suggesting that knowledge of melodies depends at least in part on declarative memory. This, in turn, has further implications. First of all, it suggests that, like language, music cognition may depend on general-purpose brain systems. We emphasize, however, that portions of these systems could become subspecialized for aspects of music cognition, both evolutionarily and developmentally, as has been suggested for language (Ullman, 2004, 2016).

Importantly, because declarative memory has been well studied at multiple levels (including its behavioral, computational, neuroanatomical, physiological, cellular, molecular, genetic, and pharmacological correlates), this vast independent knowledge about the memory system could also pertain to music cognition (Ullman, 2004, 2016). Thus, as with language, linking music cognition to declarative memory could generate a wide range of novel predictions that there might be no independent reason to make based on the more circumscribed study of music cognition alone (Ullman, 2016). For example, the anatomical, developmental and genetic correlates of declarative memory might also be expected to underlie music, in particular ways. An understanding of the dependence of music cognition on declarative memory may therefore provide important insights regarding the evolution and development of music cognition. Overall, linking music to declarative memory could prove to be a powerful approach that may lead to substantial advances in the understanding of the neurocognition of music. These advances could include efforts to understand how knowledge about specific melodies contributes to the development, within the brains of listeners, of musical expectations. Such an understanding is crucial to the effort to understand how music is able to evoke powerful emotions and pleasure in listeners.

Linking aspects of music cognition to declarative memory could also help clarify commonalities between the cognition of music and language. Unlike proposals that have suggested that music cognition has ‘piggybacked’ on language circuitry (e.g., Pinker, 1997), here we suggest that the language/music neurocognitive commonality lies at least in part with declarative memory (also see Miranda and Ullman, 2007). On this view, this general-purpose system may underlie the cognition of both language and music, rather than music cognition depending directly on language circuitry. Of course, such a common dependence on declarative memory does not preclude any additional ‘piggybacking’ of music cognition on language (or vice versa) – either in portions of declarative memory that have evolutionarily or developmentally become specialized for language, or in any additional circuitry that might be specific to language (Ullman, 2004, 2016). Moreover, a joint language/music dependence on declarative memory does not preclude any additional joint dependence on other brain systems, including working memory and procedural memory (Miranda and Ullman, 2007).

From the perspective of memory systems, the findings presented here and in Miranda and Ullman (2007) underscore the view that declarative memory seems to underlie a wide range of types of knowledge, functions, domains, and modalities, and is not limited to episodic (event) and semantic (fact) knowledge as has traditionally been suggested (for discussion, see Ullman and Pullman, 2015; Ullman, 2016).

From a language perspective, the findings of the present study underscore the plausibility that highly specialized areas of knowledge, which are moreover found across human cultures, may depend importantly on general-purpose brain systems. This underscores the plausibility of the reliance of language on declarative memory and other general-purpose cognitive systems (Ullman, 2004, 2016).

The findings also have important implications for the study of sex differences. They reveal, for the first time, that women seem to have an advantage at recognizing familiar melodies, as compared to men. The findings also show for the first time that there are behavioral sex differences in higher-level aspects of music cognition. Importantly, the observed female superiority does *not* seem to be due to an exclusively verbal advantage, since the female advantage did not interact with lyricness. This not only strengthens the evidence of an overall female advantage at tasks involving declarative memory, and evidence of its extension to the domain of music, but also crucially throws doubt on the claim that the female advantage at many verbal tasks is specific to the verbal domain. Rather, many if not most of the previously observed female advantages at verbal tasks may instead be partly if not largely due to female advantages in declarative memory (Ullman et al., 2008). This controversial issue seems to warrant further research.

The findings of the present study also have educational and clinical implications. Pedagogical techniques that have been shown to improve learning and retention in declarative memory, such as spaced presentation and the testing (retrieval practice) effect (Cepeda et al., 2006; Roediger and Butler, 2011; Ullman and Lovelett, under review) may also be expected to enhance music learning, in particular the learning of specific melodies, just as they seem to enhance language, in particular the learning of words (Ozemir et al., in preparation; Ullman and Lovelett, under review). Also, understanding the neural substrates of the learning of knowledge about specific melodies could help guide music therapy, an approach that has been shown to be effective in helping patients with conditions involving deficits of both language and memory, such as aphasia and Alzheimer’s disease (Norton et al., 2009; Ueda et al., 2013).

### Limitations and Future Studies

This study has various limitations. Perhaps most importantly, it does not *directly* tie the observed sex differences in melody recognition to female advantages in declarative memory. Thus, some other factor or factors could at least partially account for the findings. For example, it is possible that females generally make quicker decisions than males regarding information on which confidence is not high, or that sex differences in other aspects of music cognition involved in melody recognition could lead to the observed findings.

However, the sex differences found here were *predicted* on the basis of independent findings of sex differences in declarative memory, and moreover, analyses suggested they were not due to a wide range of potentially confounding factors or alternative explanations. Additionally, previous evidence has linked knowledge of familiar melodies to declarative memory (Miranda and Ullman, 2007). Together, this suggests that the study provides initial support for the view that the female advantage at declarative memory extends to music cognition, and can at least partly explain the observed sex differences in melody recognition.

Importantly, the findings constitute a useful foundation for future studies to more directly examine the issue. For example, further studies might examine whether participants’ ability at melody recognition correlates with their ability at various declarative memory tasks. One could also examine the neural underpinnings of the observed sex differences, for example with fMRI or ERPs. Further research should also probe how broadly the apparent female advantage might hold, for example across different musical systems (e.g., in the Javanese or North Indian classical musical systems), age groups, and so on. One might also examine whether the female advantage would also hold in the actual identification of melodies (as in the game show “Name that Tune”), or whether it might be limited to binary familiarity judgments. Given the importance of sex hormones on cognition, including declarative memory (Hausmann et al., 2000; Hausmann, 2005; Ullman et al., 2008), the influence of estrogen and other sex hormones, and their variability throughout the menstrual cycle, also warrant investigation. For example, further studies may examine whether the findings obtained here might be due in part to elevated levels of estrogen during particular points along the menstrual cycle. The possibility of cultural influences (Hoffman et al., 2011) on the observed sex differences should also be investigated. Although the control task examined very simple aspects of auditory processing (i.e., the participants heard various tones and responded with a simple key press to any tone), the task did not directly examine pitch processing (since the same response was made to any pitch), nor other aspects of auditory processing such as rhythm. Futures studies could control for such aspects of auditory processing, for example with different responses for different pitches or rhythms. It would additionally be highly informative to examine the *learning* of new specific melodies, and whether and how this depends on declarative memory. Finally, future studies might extend the investigation of music cognition to procedural memory, to examine whether and how the learning or use of musical syntax, or other aspects of music, might depend on this system.

## Conclusion

This study revealed, for the first time, a female advantage at recognizing familiar melodies, as compared to males. This pattern, which showed a large effect size, held across musicians and non-musicians, and over melodies with and without commonly associated lyrics. We predicted the female advantage based on independent evidence suggesting both a female advantage at declarative memory and a dependence of knowledge of familiar melodies on this system. Although some caution is warranted because this is the first study to examine sex differences in melody recognition, the findings lend support to the hypothesis that knowledge pertaining to specific melodies indeed depends on declarative memory, which in turn leads to a female advantage at familiar melody recognition, thanks to a more general female advantage at declarative memory. The finding that the female advantage held across melodies that are and are not associated with lyrics argues against the view that the commonly observed female advantage at tasks involving verbal (or verbalizable) material is best explained by a sex difference specific to the verbal domain. Additionally, because declarative memory also underlies language, it seems likely that the cognitive commonalities between music and language may be explained, at least in part, by a common dependence on declarative memory. More generally, because declarative memory is well studied at many levels, evidence that aspects of music cognition rely on this system could lead to a powerful research program capable of generating a wide range of novel predictions for the neurocognition of music.

## Author Contributions

All authors listed, have made substantial, direct and intellectual contribution to the work, and approved it for publication.

## Conflict of Interest Statement

The authors declare that the research was conducted in the absence of any commercial or financial relationships that could be construed as a potential conflict of interest.
