# Supplementary material for: Sex Differences in Music: A Female Advantage at Recognizing Familiar Melodies
Source: Front Psychol. 2016 Mar 1;7:278. doi: 10.3389/fpsyg.2016.00278 (PMC4771742; doi:10.3389/fpsyg.2016.00278)
Supplement: Supplementary file 1 [file Data_Sheet_1.DOCX]

Appendix: The 130 Familiar Melodies

| 12 Days of Christmas | Hush Little Baby | Row Row Row Your Boat |
| --- | --- | --- |
| 76 Trombones | I Feel Pretty | Rudolph the Red Nosed Reindeer |
| 99 Bottles of Beer on the Wall | I Heard the Bells on Christmas Day | Schindler's List |
| Addams Family | I'm a Little Teapot | Silent Night |
| Amazing Grace | I'm Dreaming of a White Christmas | Silver Bells |
| America | Imperial March from Star Wars | Simpsons |
| America the Beautiful | Inspector Gadget | Skip to My Lou |
| Anchors Aweigh | It's a Small World | Someone's in the Kitchen with Dinah |
| Andy Griffith | It's Beginning to Look a lot Like Christmas | Spoonful of Sugar - Mary Poppins |
| Angels We Have Heard on High | Itsy Bitsy Spider | Swan Lake |
| Are You Sleeping (Where is Thumbkin) | I've Been Working on the Railroad | Tango |
| Auld Lang Syne | Jeopardy | Ta-ra-ra Boom-der-e |
| Baby Bumble Bee | Jesus Loves Me | Tetris |
| Back to the Future | Jingle Bells (Chorus) | The Christmas Song (Chestnuts Roasting) |
| Battle Hymn of the Republic | John Jacob Jingleheimer Schmidt | The Entertainer |
| Caisson Song | Jolly Old Saint Nicholas | The First Noel |
| California Girls | Joyful Joyful We Adore Thee | The Lion Sleeps Tonight |
| Chariots of Fire | Jupiter - Holst | The Rose |
| Clementine | Jurassic Park | The Song That Never Ends |
| Coca-Cola | Let It Snow | This Land is Your Land |
| Colonel Bogie | London Bridge is Falling Down | This Old Man (Barney Song) |
| Crimson Tide | Long Long Ago | Three Blind Mice |
| Deck the Halls | Lullaby | Tis a Gift to be Simple |
| Ding Dong, the Witch is Dead | Mary Had a Little Lamb | Top Gun |
| Dixie | Minuet (Bach) | Toreador March from Carmen |
| Do Your Ears Hang Low | Muffin Man | Turkey in the Straw |
| Farmer in the Dell | My Hat, It Has Three Corners | Twinkle Twinkle Little Star |
| Flintstones | National Anthem | Unknown |
| Frosty the Snowman | NBC News | Up on the Housetop (Intro) |
| Gilligan's Isle | Oh Come, All Ye Faithful | US Marines |
| Go Tell it on the Mountain | Oh, Christmas Tree | Washington Post March |
| God Bless America | Oh, Holy Night | Water Music (Hornpipe) |
| God Bless the USA | Oh, Little Town of Bethlehem | We Wish You a Merry Christmas |
| Godfather | Oh, Suzannah | West Side Story - America |
| Good King Wenceslas | Old MacDonald | What Child is This |
| Habanera from Carmen | Oscar Meyer Weiner | Wheels on the Bus |
| Hail to the Chief | Pomp and Circumstance | When the Saints Go Marching In |
| Happy Birthday | Pop Goes the Weasel | When You Wish Upon a Star |
| Hark the Herald Angels Sing | Praise God (Doxology) | Winnie the Pooh |
| Have a Holly Jolly Christmas | Puff the Magic Dragon | Yankee Doodle |
| Heart 'n Soul | Raiders March from Indiana Jones | You Are My Sunshine |
| Here Comes Santa Claus | Rock-a-bye Baby | You're a Grand Old Flag |
| He's a Jolly Good Fellow | Rockin' Around the Christmas Tree | Zip-a-dee-doo-dah |
| How Much is that Doggie in the Window |  |  |
